# Building partnerships, capacity, and knowledge through a use of newly linked child development and education datasets in Ontario, Canada.

**DOI:** 10.23889/ijpds.v7i3.1942

**Published:** 2022-08-25

**Authors:** Magdalena Janus, Jeanne Sinclair, Jennifer Hove, Scott Davies

**Affiliations:** 1 Public Health Scotland; 2 National Records of Scotland; 3 Public Health Scotland; 4 National Records of Scotland

## Objectives

The objective of this study was to establish a partnership between a university and a jurisdictional education body (Education Quality and Assessment Organization, EQAO) which would allow creation of a linked dataset from kindergarten to later grades in order to examine educational trajectory in mathematics in Ontario.

## Approach

Building on mutual goals of improving the understanding of children’s learning trajectories, we developed a project with an investigator team that included university researchers and representatives of the provincial educational assessment body, to link a database of child development status in kindergarten (Early Development Instrument/EDI data, including neighbourhood socioeconomic/SES index) with academic assessment EQAO data, and received research funding. A deterministic matching process was employed to match the datasets. We examined differences between the unmatched and fully matched cases and constructed a growth mixture model of math scores in grades 3, 6 and 9, with key EDI/SES variables as covariates.

## Results

Despite lacking a common identifier, we successfully matched approximately 50% of the EDI cases from 2002-2014 (n=183,771). Effect sizes indicated negligible differences between matched and unmatched, except for SES and child development status, which were poorer for unmatched group. A 3-class solution was the best fit for a 20,000-person subsample of math trajectories based on AIC, BIC, ICL, and entropy values as well as sufficiently high proportions of posterior probabilities, which indicate confidence in class membership. 61% of sample showed steady moderate-high achievement; 9% started high, but declined, and 30% deteriorated then improved. Males, children in low SES, and those with adequate kindergarten EDI outcomes had better math achievement trajectories than females, children in high SES, and those with poor kindergarten outcomes.

## Conclusion

Given the two datasets were collected without explicit linkage plan, the matching was only 50%, nevertheless resulting in a large database that allows study of early development antecedents of students’ educational trajectories. The partnership between university and EQAO ensures a wide dissemination of results in both academia and policy worlds.

